# Assessing inequality, irregularity, and severity regarding road traffic safety during COVID-19

**DOI:** 10.1038/s41598-021-91392-z

**Published:** 2021-06-23

**Authors:** Lei Lin, Feng Shi, Weizi Li

**Affiliations:** 1 Goergen Institute for Data Science, University of Rochester, Rochester, USA; 2 Odum Institute for Research in Social Science, University of North Carolina at Chapel Hill, Chapel Hill, USA; 3 Department of Computer Science, University of Memphis, Memphis, USA

**Keywords:** Computational science, Public health

## Abstract

COVID-19 has affected every sector of our society, among which human mobility is taking a dramatic change due to quarantine and social distancing. We investigate the impact of the pandemic and subsequent mobility changes on road traffic safety. Using traffic accident data from the city of Los Angeles and New York City, we find that the impact is not merely a blunt reduction in traffic and accidents; rather, (1) the proportion of accidents unexpectedly increases for “Hispanic” and “Male” groups; (2) the “hot spots” of accidents have shifted in both time and space and are likely moved from higher-income areas (e.g., Hollywood and Lower Manhattan) to lower-income areas (e.g., southern LA and southern Brooklyn); (3) the severity level of accidents decreases with the number of accidents regardless of transportation modes. Understanding those variations of traffic accidents not only sheds a light on the heterogeneous impact of COVID-19 across demographic and geographic factors, but also helps policymakers and planners design more effective safety policies and interventions during critical conditions such as the pandemic.

## Introduction

The coronavirus disease 2019 (COVID-19) has undoubtedly impacted all aspects of our society^1,2^. In particular, the human mobility is taking a big hit due to quarantine and social distancing. For example, the total amount of travels in the US dropped 71% between early March and mid April^3^ in 2020. A direct consequence of the reduced mobility is reduced traffic, and a seemingly straightforward question follows: How is road safety affected by the COVID-19 pandemic? This simple question turns out to have conflicting answers.

On one hand, reduced mobility leads to a decreased number of traffic accidents because the number of accidents is known to be positively correlated with the amount of traffic^4^. A reduction of almost 50% in traffic accidents after the stay-at-home orders is found in five states in the US^5^. On the other, researchers and authorities both find that the new traffic pattern can cause frequent speeding, careless driving, and even “revenge driving”, hence worsening road safety. One report from the National Safety Council (NSC) shows that the fatality rate of traffic accidents increases 14% in March 2020, compared to the same period in 2019^6^, while others find the number of fatalities either increasing or decreasing in different regions due to various factors including topography, driving culture, or police recording procedures^7^.

Previous studies have mainly taken the descriptive approach to analyze road safety and used government orders’ dates as the mobility change-point^5,8,9^. Studies that statistically evaluate the road safety during the pandemic and use systematically-determined mobility change-point are scarce. Besides, previous studies mainly examine the temporal changes of road safety, changes of the spatial distribution of traffic accidents are largely under-investigated. Here we take a systematic and statistical approach to assessing road traffic safety during the COVID-19 pandemic. Using traffic accident data from the city of Los Angeles (LA)^10^ and New York City (NYC)^11^, we find that although the number of accidents drops substantially across demographic groups during the pandemic, different groups are affected disproportionately. Beyond demographics, we also observe that the “hot spots” of accidents have shifted in both time and space. Finally, we find one positive change during the pandemic—the severity level of accidents decreases with the number of accidents. Overall, the impact of COVID-19 on road traffic safety is found far more complicated than a blunt reduction in traffic and accidents.

Understanding traffic accidents in various demographic groups will not only shed a light on the heterogeneous impacts of COVID-19 but also contribute to transportation inequity analysis in general^9^. Road traffic safety has been a long-standing challenge for modern society, with an estimate cost of $871 billion dollars annually in the U.S.^12^. In order to contain the spread of COVID-19, a natural experiment is conducted as a byproduct that dramatically reduces the traffic and provides unprecedented data to study how traffic accidents change accordingly. Although such measures are not reproducible, understanding their effect can help us design more effective road safety policies and interventions.

Worth noting, existing studies have analyzed the impact of COVID-19 on various aspects of our transportation systems, including transport energy^13^, air pollution^14^, and freight transportation^15^. Researchers have also explored the potential of redesigning buildings and urban areas, such as leveraging sustainable built environment solutions, to prevent the spread of COVID-19^16^. We contribute to this broad literature by filling the gap of the impact of COVID-19 on road traffic safety.

## Results

### Mobility change-point

**Figure 1:**
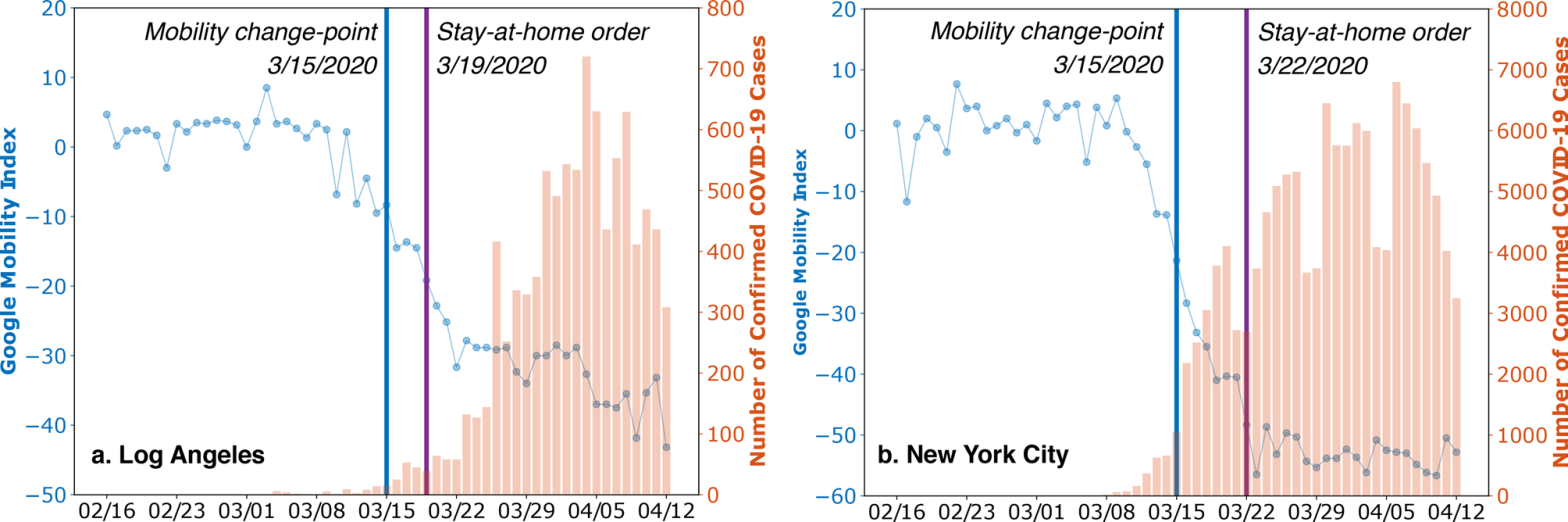
Detected mobility change-points, Stay-at-Home order dates, daily new COVID-19 cases, and Google Mobility Index of LA and NYC are shown.

The mobility change-point is the date when there is an abrupt, dramatic change of the mobility flow of a large group of people (See Methods for detailed definitions). Existing studies mainly use the dates of government’s stay-at-home orders as the mobility change-points^5,8,9^, ignoring the fact that the society might have different reaction time to the disease^17^. Here, we resort to a systematic and statistical approach via the change-point detection algorithm^18^. The mobility time series is chosen to be the Google Mobility Index^19^. Figure 1 shows the detected mobility change-point, March 15, 2020, which is prior to the dates of stay-at-home orders (e.g., March 19 and March 22). Using this date, we study the differences in traffic accidents beforehand and afterwards.

### Inequality

**Figure 2:**
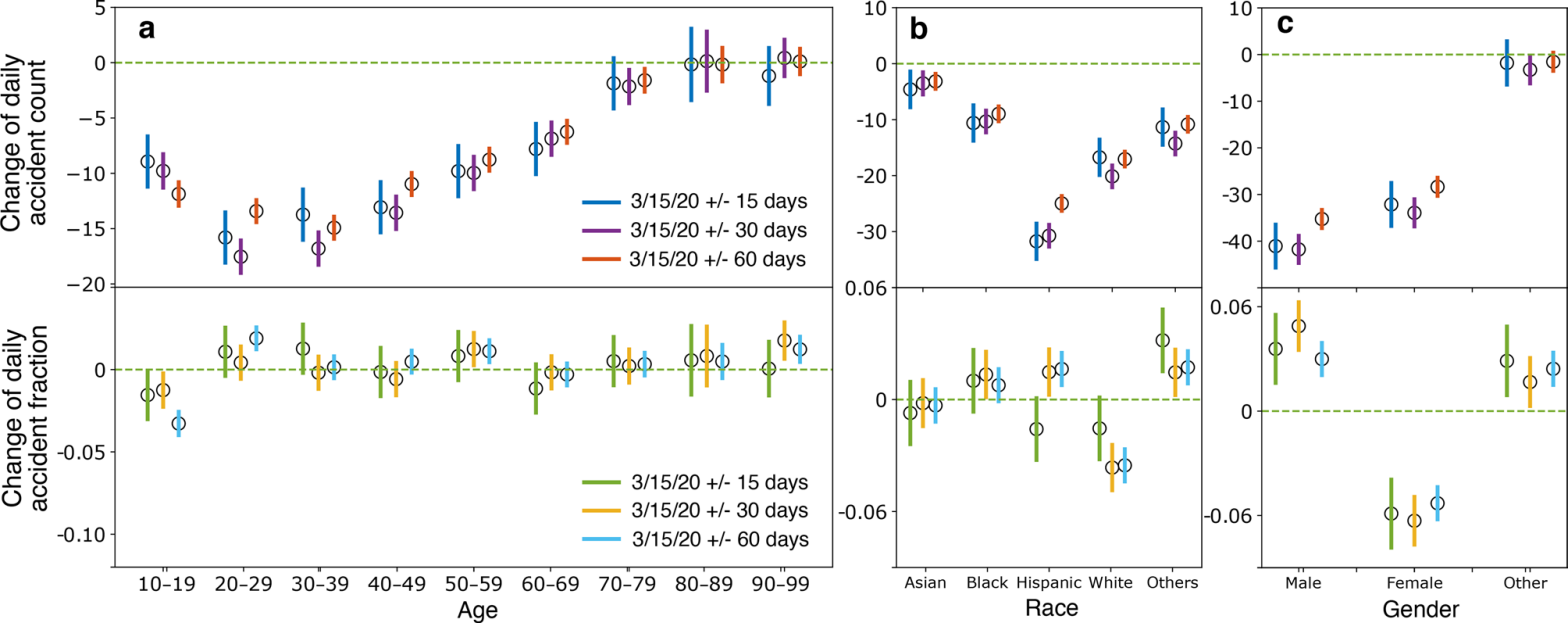
Change of daily accidents across age, race, and gender groups. Top: Change of daily accident count. Bottom: Change of daily accident fraction (the fraction of daily accidents among all accidents that day for each group). 95% confidence intervals are shown as vertical bars for each data point. Also shown are estimates from three time windows: 15 days (blue and green), 30 days (purple and orange), and 60 days (red and cyan) before and after the mobility change-point.

Figure 2 shows the change of daily traffic accidents concerning age, race, and gender before and after the mobility change-point. The top row presents the changes in daily accident counts: they are significantly negative in most demographic groups (meaning the number of accidents decreases in general). However, the share of accidents of each demographic group (i.e., the fraction of accidents among the total number of accidents for each group) shows a different pattern in the bottom row of Fig. 2. These results highlight the unequal impact of the pandemic on different demographic groups. To assess the robustness of the results in time, we consider three time windows: 15 days, 30 days, and 60 days, before and after the mobility change-point (same for all other analyses unless stated otherwise). We remove seasonal effects from the changes using the difference-in-differences method detailed in the Methods section.

Specifically, all age groups except “70–79”, “80–89”, and “90–99” have significant reductions in daily accident counts. The groups “20–29” and “30–39” have the largest decrease, but their shares of the accidents do not change significantly. In fact, only the age group “10–19” has a significant (although barely) decrease of 3.2% in the share of accidents. Overall, the pandemic does not seem to have a largely biased impact across age groups.

Regarding race, all groups experience a significant reduction in accident counts. The “Hispanic” group has the largest decrease, but its share of accidents actually increases after a transient reduction in the first 15 days. In comparison, the “White” group has a significant reduction in both measures.

Regarding gender, both “Male” and “Female” groups have significant reductions in the number of accidents. However, in terms of the share of accidents, the “Male” group has increased around 4%, while the “Female” group has decreased around 5%. In sum, the distribution of accidents has been shifting its mass towards “Hispanic” and “Male” groups, not because the increase of their numbers of accidents but the disproportionate decrease of accidents across the groups.

We have further attempted to assess the intersectionality between gender, race, and age. A linear regression on gender and race and their interactions: $$y\sim gender*race*period$$, where *y* is daily accident count and $$*$$ means all interactions, is conducted. However, due to data scarcity in covering all interactions (of all factors), only “Hispanic$$*$$Female” witnesses a significant decrease in daily accident count ($$p=0.003$$) during the pandemic. While changes of other groups are insignificant, the results agree with our findings in terms of statistical trend. For example, we find that “Hispanic$$*$$Male” has the largest drop, followed by “Hispanic$$*$$Female” and “White$$*$$Male”, agreeing with the patterns in Fig. 2

### Irregularity

**Figure 3:**
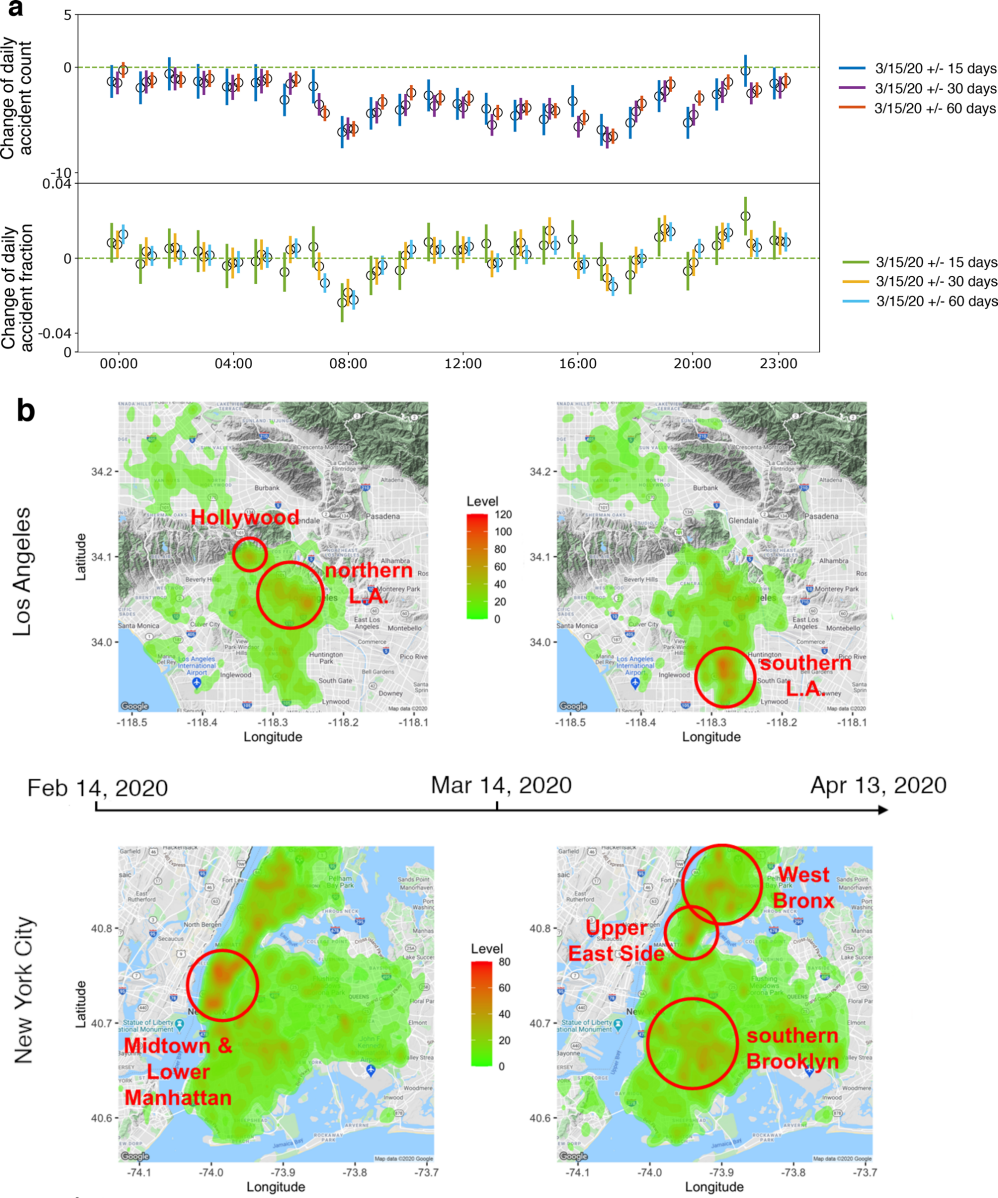
Temporal and spatial shifts of accident hot spots. (**a**) Change of daily accident counts (top) and fractions (bottom) grouped by different hours of a day in NYC. 95% confidence intervals are shown as vertical bars for each data point. Also shown are estimates from three time windows: 15 days (blue and green), 30 days (purple and orange), and 60 days (red and cyan) before and after the mobility change-point. (**b**) Heatmap of traffic accidents in LA (top) and NYC (bottom) between Feb. 14, 2020 and Apr. 13, 2020, using Mar. 15 2020 as the mobility change-point. The maps are produced by R package *ggmap* 3.0.0 (github.com/dkahle/ggmap) and Google Map service (cloud.google.com/maps-platform/).

Figure 3a shows the change of accidents at different hours of a day. The hours between 06:00 and 22:00 have seen significant decreases in accident counts. Especially during the morning (08:00) and afternoon (17:00) rush hours, not only the accident counts but also the shares of accidents decrease significantly. As a result, the accidents are re-distributed throughout the day with a significant increase in share during 19:00, demonstrating temporal irregularity.

To examine the spatial distribution of accidents during the pandemic, we perform a kernel density estimation^20^ on the accident locations during the 30-day periods before and after the mobility change-point. Figure 3b shows the heatmaps of the estimated kernel densities. There are two hot spots of traffic accidents in LA prior to the pandemic with as high as 80 accidents per month: one around Hollywood and the other around northern downtown LA. After the mobility change, the hot spots have shifted to southern LA, with the number of traffic accidents increased to more than 110 per month. In NYC, the accident hot spots are distributed around Midtown Manhattan and Lower Manhattan prior to the pandemic; during the pandemic, the hot spots have shifted to Upper East Side, West Bronx, and southern Brooklyn.

We also statistically compare the observed patterns with 2019 data to rule out any seasonal shift of accident locations. We conduct global two-sample tests^20^ on the kernel densities estimated from different time periods. In LA, the estimated kernel density in 3/15–4/13 in 2020 is significantly different than those in the other three periods ($p=0.004$ for 2/14–3/14 in 2020, $p=0.009$ for 3/15–4/13 in 2019, and $p=0.002$ for 2/13–3/14 in 2019), while the estimated kernel densities in 2/13–3/14 and 3/15–4/13 in 2019 are not statistically different ($p=0.44$). The pattern changes found in NYC are more significant: the estimated density in 3/15–4/13 in 2020 is significantly different from all the others with *p*-value$<0.001$, and the estimated kernel densities in 2/13–3/14 and 3/15–4/13 in 2019 are not statistically different ($p=0.11$). Using both visual inspections and statistical tests, we conclude that the shifts of accident hot spots during the pandemic are statistically significant and are not caused by seasonal effects.

### Severity

**Figure 4:**
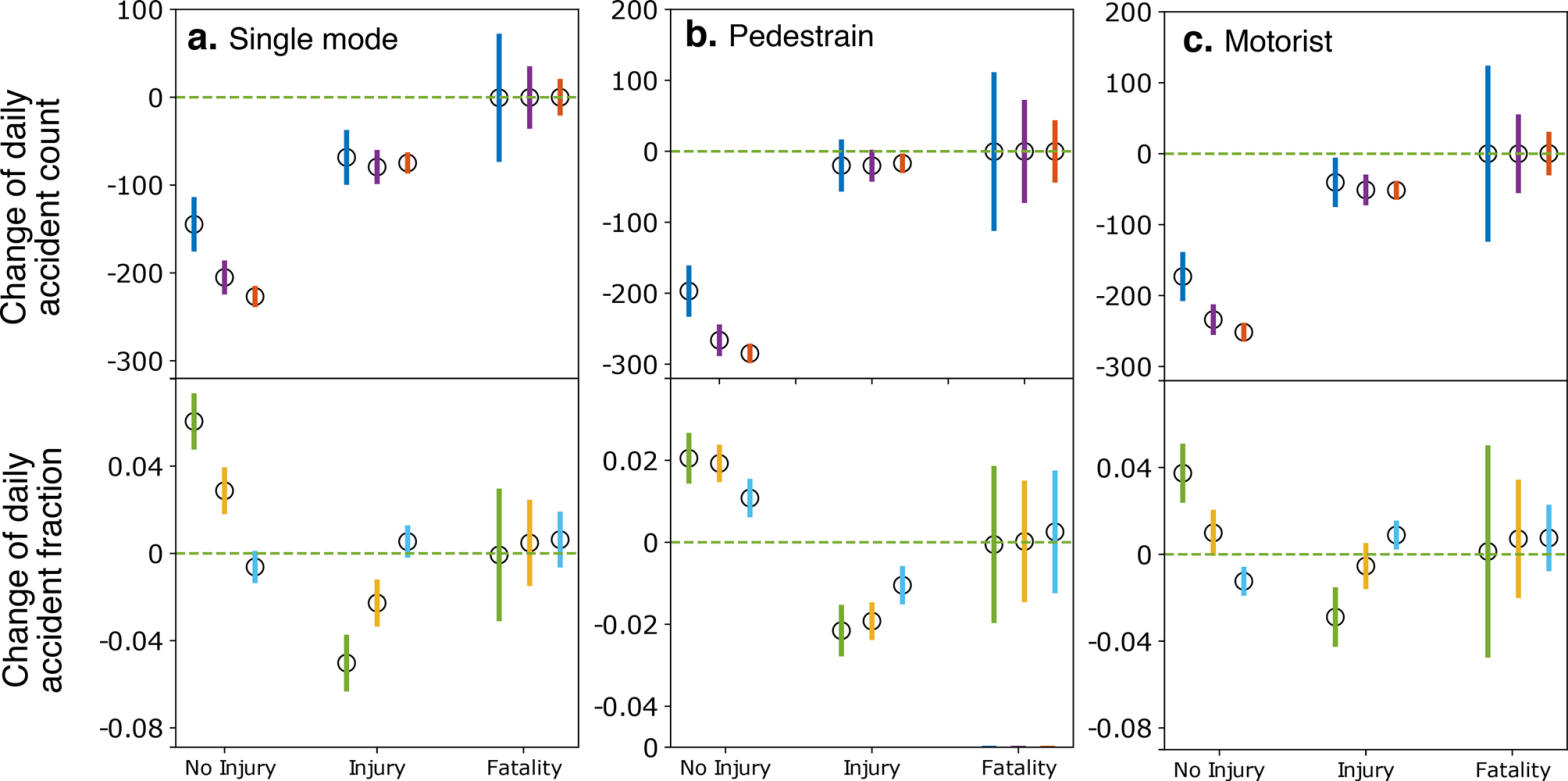
Change of daily accidents in NYC after the mobility change for different severity levels: no injury, injury, and fatality. The accidents are divided into three types: (**a**) not involving other transportation modes, (**b**) involving pedestrians, and (**c**) involving motorists. 95% confidence intervals are shown as vertical bars for each data point. Also shown are estimates from three time windows: 15 days (blue and green), 30 days (purple and orange), and 60 days (red and cyan) before and after the mobility change-point.

Figure 4 reports the changes in traffic accidents regarding severity levels. As severity levels are typically associated with transportation modes, we further divide the accidents into three types: (1) those not involving other transportation modes (single mode), (2) those involving pedestrians, and (3) those involving motorists. We find insignificant changes in fatal accidents across all three types, while the counts of accidents with and without injuries both drop significantly. What is notable is that the share of no-injury accidents increases significantly after the mobility change, implying that the distribution of accidents shifts towards “light” accidents without injuries. However, this shift appears to be reversing as we progress longer (60 days) into the pandemic. Overall, the severity level of accidents decreases (reflected by the increase of the fraction of no-injury accidents) during the pandemic.

## Discussion

**Figure 5:**
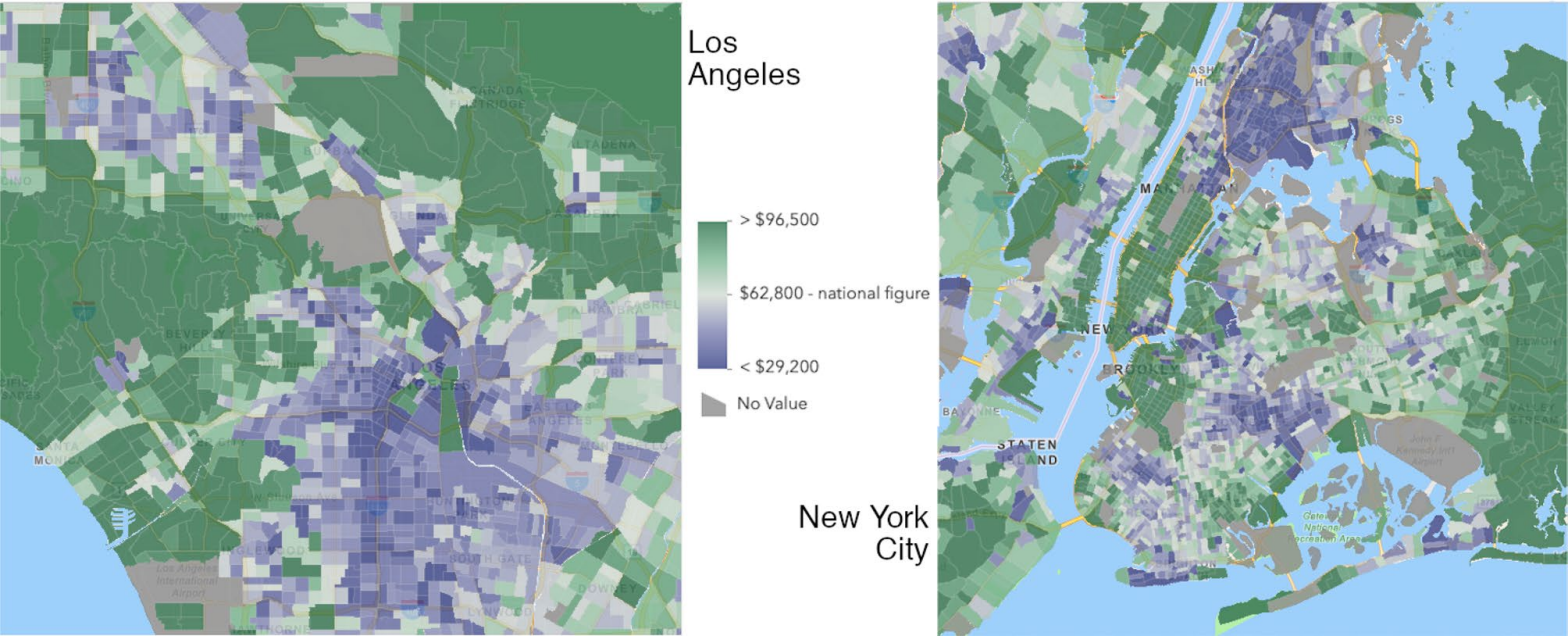
Median household income by tract in LA (Left) and NYC (Right). The income data is from American Community Survey 2019, and the maps are produced by ArcGIS Online (www.arcgis.com).

While it is as expected that the number of accidents decreases during the pandemic due to reduced mobility, we find that this reduction occurs unequally among demographic groups. First, for groups older than 70, the changes are insignificant, which may be due to that seniors in general travel less and hence are not impacted much by the mobility change. Second, the shares of accidents actually increase in “Hispanic” and “Male” groups, while the shares of other groups remain unchanged or decrease. We believe that understanding the causes of the inequality is critical, which we defer to follow-up studies. Here we hypothesize social economic status (SES) being one of the factors causing the inequality. For example, responses to social distancing are found to differ by income^21^. Accordingly, different SES groups may experience different levels of mobility change. When we compare the spatial distribution of accidents (Fig. 3b) with the map of income (Fig. 5), we find that the hot spots of accidents have shifted from higher-income areas (e.g., Hollywood and Lower Manhattan) to lower-income areas (e.g., southern LA and southern Brooklyn). This may be due to that unprivileged SES groups are constrained by their capacity to work from home, take time off of work, and live on savings^22^. Further studies are required to fully understand the relationship between SES and road traffic safety.

In this study, we find that the mobility change happens a few days earlier than governments’ stay-at-home orders at LA and NYC. This indicates public’s early response to the pandemic, agreeing with previous studies^17^, while other studies have found a delay in the mobility adjustment^2,23^. This inconsistency highlights the necessity of a data-driven approach to study road traffic safety and topics alike. Another related topic is analyzing risk and its relationship to disaster, vulnerability, and exposure. However, after scrutinizing many data sources, we cannot find one covering necessary attributes for LA and NYC. So, such analysis is omitted.

Overall, our study sheds lights on many practical aspects of reducing economic and life losses due to traffic accidents during critical conditions such as the pandemic. For example, given certain groups are experiencing relatively more accidents, more mobility resources can be allocated to those groups. We believe our study can provide some insights for social scientists to understand the found differences in the distribution of traffic accidents and for city designers and planners to better plan land and road network uses.

## Methods

### Data

Multiple datasets are used in this study. The Google Community Mobility Reports^19^ are used to detect mobility change-points. The dataset contains a daily mobility index that combines traffic at different regions including retail, recreation, grocery, pharmacy, and parks. Two datasets on traffic accidents are used in examining the change of accidents before and after the mobility change: one from LA^10^ and the other from NYC^11^. Both datasets record the time and location of accidents, which enable us to investigate the spatial-temporal patterns of accidents. The dataset of LA also contains demographic attributes (i.e., age, race, and gender) of accident victims. The dataset of NYC contains the severity level (i.e., no injury, injury, and fatality) and transportation modes (i.e., pedestrian, motorist, and no other modes) of accidents. All datasets are complete without missing data.

### Mobility change point

The mobility change-point is the date when there is an abrupt, dramatic change of the mobility flow of a large group of people (e.g., all residents in LA or NYC). There are two technical challenges in defining the mobility change-point. First, how to measure the mobility of a large group of people? And second, how to assess the level of change since changes are perpetual? We address the first challenge using the Google Mobility Index^19^. We address the second challenge using a widely-adopted technique for change-point detection in time series^18^.

Formally, the problem is defined as follows: consider a non-stationary time series $m=\{m_t\}_{t=1}^T$, which may have abrupt changes at *K* unknown time steps $1<t_1<t_2<\cdots<t_K<T$. The goal is to find these unknown time steps via solving the following optimization program:

1 $$\begin{aligned} \min _{\tau } V(\tau ) + \beta K, \end{aligned}$$

where $\beta$ is the weighting factor; $\tau = \{t_1,t_2,\ldots ,t_K\}$ represents the segmentation of the time series; $V(\tau )$ is defined as:

2 $$\begin{aligned} V(\tau ) = \sum _{k=0}^{K}c(m_{t_k}..m_{t_{k+1}}), \end{aligned}$$

where $c(\cdot )$ is the cost function that measures the similarity of elements in a time series segment.

The mobility time series is chosen to be the Google Mobility Index^19^ and $c(\cdot )$ is chosen to be the radial basis function with the default settings in the Python package ruptures^18^.

### Difference-in-differences analysis

We take the difference-in-differences (DID) regression^24^ to statistically test the change of traffic accidents. DID is a standard approach to control for seasonal effects when dealing with long-term periods. For example, the difference in daily accident counts before and after March 15, 2020 could be attributed to the seasonal change from Winter to Spring. To rule out this possibility so that we can study the effect of COVID-19 and lockdown, we compare accident data in 2019 and 2020 over the same time periods.

To be specific, each accident has two basic features: year and period. *year* is a categorical variable taking one of the two values: 2019 or 2020; *period* is also a categorical variable taking of of the two values: before or after the mobility change-point. As we are interested in the distribution of accidents across various demographic factors, we consider the third feature which takes values such as age, gender, race, or severity level. We then aggregate daily accidents for each  combination of the three features and denote *y* the accident counts. Lastly, we construct the following linear regression model to test the change of accidents (using gender as the third feature):

3 $$\begin{aligned} y \sim year*period*gender, \end{aligned}$$

where $$*$$ denotes all possible interactions. $$year*period*gender$$ contains not only individual variables but also their two-way and three-way interactions, i.e., $$year + period + gender + year \times period + \cdots + year \times period \times gender$$. Following the convention of categorical variable analysis, the independent variables are converted into dummy variables.

The quantity of interest here is the coefficients of three-way interactions, which give the difference in daily accident counts before and after the mobility change for different values of the third feature (e.g., gender). A positive (negative) coefficient suggests an increase (decrease) of accidents with the value indicates the magnitude of change. Confidence intervals are also calculated to show the statistical significance.

As the number of accidents is found to correlate with the amount of traffic and decrease after the mobility change in general, we further investigate whether the number of accidents changes disproportionately across all factors. This is done by replacing the dependent variable *y* in Eq. (3) with the daily fraction of accidents in a group (e.g., Male). We then estimate the same regression model to get the change in accident fraction or share of accidents for each group.

To assess the robustness of the results in time, we fit the regression models to three time windows (separately): 15 days, 30 days, and 60 days before and after the mobility change-point. This allows us to study the temporal evolution of the changes of accidents. For example, it is possible that the number of accidents decreases (of a certain group) in the first 15 days after mobility change but recovers after 30 days.
